# Psychometric Evaluation of the Turkish Version of the Induction Compliance Checklist in Children Undergoing Mask Anesthesia

**DOI:** 10.1002/pan.70290

**Published:** 2026-08-19

**Authors:** Yesim Andiran Senayli, Nida Kiyici, Sevgi Ulusoy Tangul, Cevdet Yardimci, Mehmet Kara, Atilla Senayli, Zeev N. Kain

**Affiliations:** ^1^ Department of Anesthesiology and Reanimation Yozgat Bozok University Yozgat Turkiye; ^2^ Department of Medical Services and Techniques Yozgat Bozok University Yozgat Turkiye; ^3^ Department of Pediatric Surgery Yozgat Bozok University Yozgat Turkiye; ^4^ Department of Anesthesiology and Perioperative Care University of California Irvine California USA

**Keywords:** behavior observation techniques, child, general anesthesia, inhalation anesthesia, preoperative care, preschool child, psychometrics

## Abstract

**Background:**

Many children undergoing anesthesia commonly exhibit distress behaviors such as anxiety, crying, and physical resistance. Assessment of this behavior is important as it could lead to postoperative behavioral changes and delayed recovery.

**Aim:**

The aim of this study was to evaluate the content validity, construct validity, internal consistency, and inter‐observer reliability of the Turkish version of the Induction Compliance Checklist (ICC), which was developed to assess compliance behaviors in children during anesthesia induction.

**Methods:**

The study was planned as a prospective observational study. Following translation of the original ICC, content validity was examined using the Davis technique with the opinions of 10 experts. Next, Turkish ICC was applied simultaneously and independently by three independent observers during mask induction to 184 children (ASA I‐III) aged 1–7 years undergoing anesthesia and surgery. Inter‐observer reliability was evaluated with Intraclass Correlation Coefficient (ICC) and Fleiss Kappa/Gwet's AC1; internal consistency with KR‐20 and McDonald's omega; and construct validity with single‐factor confirmatory factor analysis (WLSMV). Item analysis was performed with a tetrachoric correlation matrix, floor and ceiling effects.

**Results:**

Content validity was excellent (I‐CVI 0.90–1.00, S‐CVI≈0.99). Inter‐observer reliability for the total score was excellent: InCC (1, 2) = 0.92 (95% CI: 0.90–0.94), InCC (2, 3) = 0.97 (95% CI: 0.96–0.98). At the item level, Gwet's AC1 values ranged from 0.81 to 0.97, while Kappa values were generally 0.75–0.90 except for M11. Internal consistency was at an acceptable level with KR‐20 0.49–0.52 and McDonald's omega 0.66–0.82. These results are low due to the floor effect caused by premedication. Confirmatory factor analysis moderately supported the single‐factor structure (best fit in Observer Surgeon: CFI = 0.927, TLI = 0.907). High floor effect was prominent especially in rarely observed severe behaviors (M7‐M11); strong correlation was observed between M9‐M10 (0.89–0.94).

**Conclusions:**

Following rigorous analysis, we found that the Turkish ICC is a reliable and valid tool that can assess children's compliance behaviors during pediatric anesthesia induction. Excellent inter‐observer agreement and high content validity support its suitability for clinical and research use. The Turkish Induction Compliance Checklist is a reliable and valid measurement tool in the Turkish pediatric population. It can contribute to standardized evaluation of child behaviors during anesthesia induction and help improve the quality of perioperative care.

## Clinical Implications

**What Is Already Known About the Topic:**

The Induction Compliance Checklist (ICC) is a validated English‐language tool for assessing observable behavioral compliance during pediatric mask induction, but no psychometrically evaluated Turkish version has previously been available.

**What New Information This Study Adds:**

This study provides a reliable and content‐valid Turkish version of the ICC (T‐ICC) with excellent inter‐observer agreement. The scale can now be used for standardized assessment of induction compliance in Turkish‐speaking pediatric populations, supporting both clinical monitoring and future intervention studies.

## 1 Introduction

Anesthesia induction in pediatric surgical procedures is one of the most stressful stages in children undergoing surgery [1, 2]. During mask induction, a significant number of children may exhibit distress behaviors such as fear, anxiety, crying, physical resistance, or passivity [2, 3, 4, 5, 6, 7, 8, 9, 10]. These negative behaviors are associated with long‐term consequences such as postoperative pain, emergence delirium, negative behavioral changes, and decreased family satisfaction [1, 8, 9].

Objective assessment of children's behavior during anesthesia induction is important for evaluating preoperative interventions, including anxiolytic premedication and parental presence, and for improving perioperative clinical practice [1]. However, anxiety and compliance during induction of anesthesia in young children represent related but distinct constructs. Instruments such as the modified Yale Preoperative Anxiety Scale (m‐YPAS), the State–Trait Anxiety Inventory for Children (STAIC), and visual analog anxiety ratings primarily assess anxiety, while the Induction Compliance Checklist (ICC) was developed to assess observable compliance behaviors during induction, including cooperation, crying, resistance, and physical restraint [6, 11, 12, 13, 14].

The original ICC, developed in English by Kain et al., is an established instrument for assessing compliance behaviors during pediatric anesthesia induction and has previously undergone reliability and validity testing [1, 3, 6]. Because the ICC was developed in another language and cultural context, its psychometric properties cannot be assumed to generalize to Turkish‐speaking populations. The absence of a validated Turkish version may limit assessment of induction compliance in pediatric anesthesia studies conducted in Turkey and may contribute to variability in measured outcomes. Therefore, this study aimed to translate and culturally adapt the ICC into Turkish and to evaluate the psychometric properties of the Turkish version of the Induction Compliance Checklist (T‐ICC).

We hypothesized that the T‐ICC would demonstrate acceptable content validity, construct validity, internal consistency, and inter‐observer reliability. We submit that developing a valid and reliable Turkish version of the ICC will support objective assessment of children's compliance behaviors during anesthesia induction and provide a foundation for future studies evaluating perioperative anxiety‐management interventions in Turkish pediatric populations.

## 2 Materials and Methods

### 2.1 Ethics Statement

The study was approved by the Yozgat Bozok University Non‐Interventional Clinical Research Ethics Committee (GOKAEK) on 05.09.2024 with decision number 153. Informed Consent of parents was received according to the Declaration of Helsinki.

### 2.2 Content Validity

The Turkish version of the Induction Compliance Checklist (ICC), developed to evaluate children's compliance behaviors during anesthesia induction in the operating room, was examined for content validity. The original “Induction Compliance Checklist” was prepared in English and consists of one positive item (“Perfect induction: no negative behaviors, fear, or anxiety”) and 10 negative behavior items (Figure 1) [3].

**FIGURE 1:**
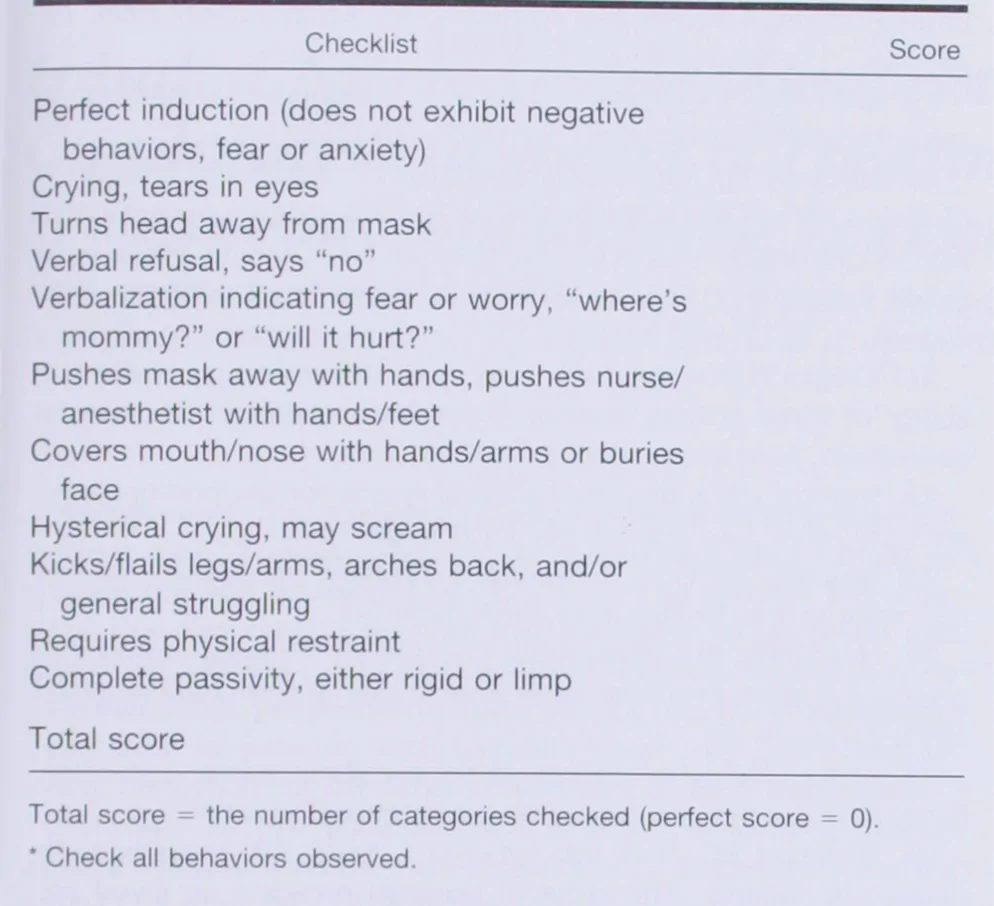
Original Induction Compliance Checklist.

The English original text was first independently translated into Turkish by two bilingual experts, both proficient in medical terminology. The two forward translations were then compared and synthesized into a single Turkish version through discussion and consensus (“cross‐referencing”), with explicit attention to semantic, idiomatic, and conceptual equivalence of each behavioral descriptor. Cultural adaptation was performed at this stage to ensure that the wording would be idiomatic and clinically meaningful in the Turkish pediatric anesthesia setting (Figure 2).

**FIGURE 2:**
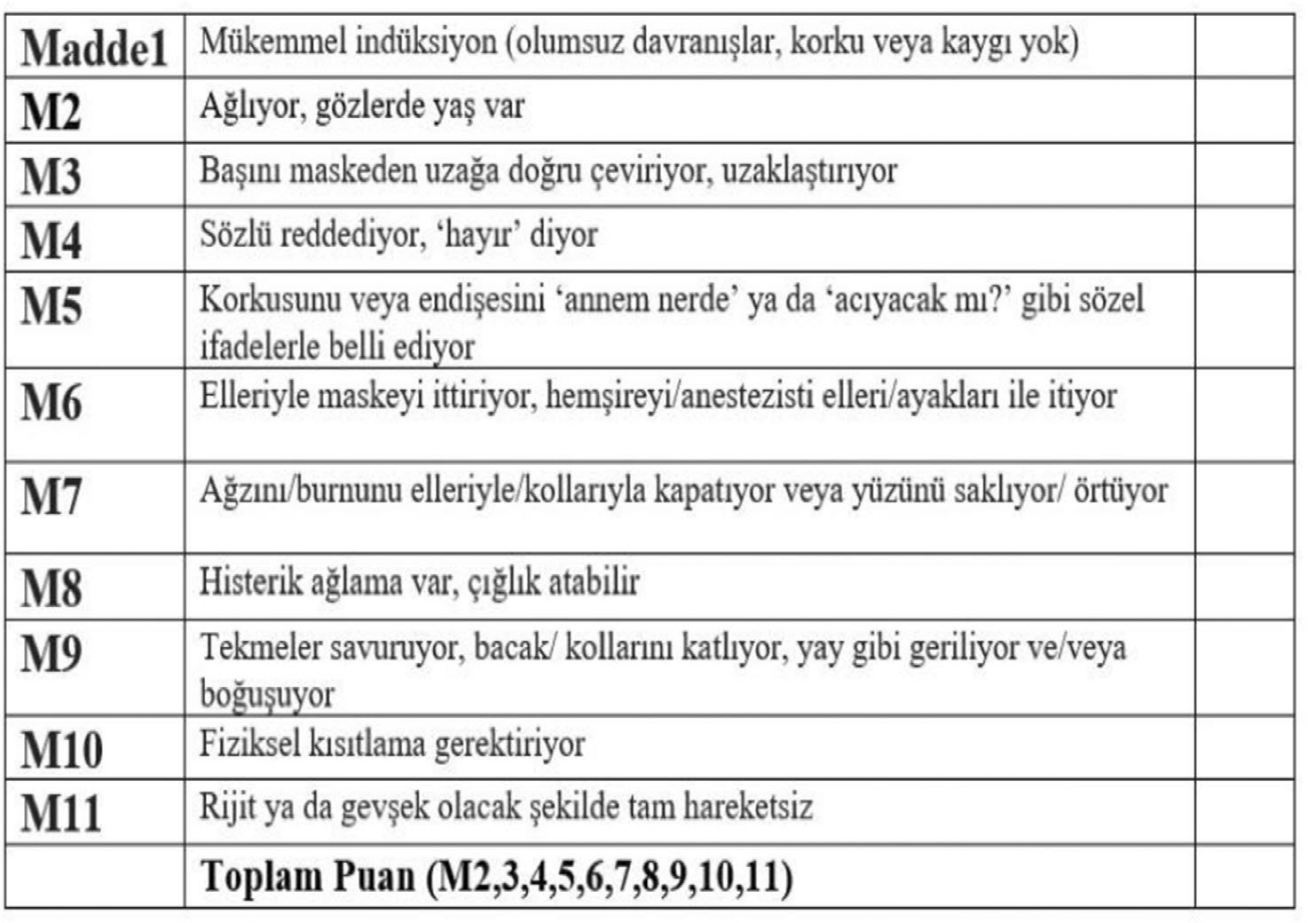
Turkish version of Induction Compliance checklist (T‐ICC).

Although a formal independent back translation was not performed, conceptual equivalence of the synthesized Turkish version to the original English instrument was subsequently verified during the content‐validity phase. Ten experts (anesthesiologists experienced in pediatric anesthesia) independently evaluated each item using the Davis technique [15] on a 4‐point Likert‐type scale (1 = not appropriate, 2 = slightly appropriate, 3 = quite appropriate, 4 = very appropriate). When calculating the Item Content Validity Index (I‐CVI), scores of 3 and 4 were considered “appropriate,” and the number of experts who rated the item as appropriate was divided by the total number of experts. The Scale Content Validity Index (S‐CVI) was calculated as the average of all I‐CVI values. An I‐CVI value of ≥ 0.80 was accepted as the item acceptance criterion [16]. The high I‐CVI (0.90–1.00) and S‐CVI (≈0.99) values obtained confirmed that the Turkish items were judged by domain experts to retain the conceptual meaning of the original scale.

#### 2.2.1 Data Collection and Evaluation Procedure

The study included 184 consecutive pediatric patients aged 1–7 years who were scheduled to undergo elective surgery and classified as ASA I–III according to the American Society of Anesthesiologists physical status classification. Patients undergoing emergency surgery, those with neurological or developmental disorders, and cases in which parental consent could not be obtained were excluded from this study. Thirty minutes before anesthesia induction, oral midazolam 0,5 mg/kg was given all children, and then anesthesia was induced with sevoflurane via mask.

Children's behaviors during anesthesia induction (mask induction with sevoflurane) were evaluated simultaneously and independently by three observers using the Turkish Induction Compliance Checklist. The evaluations were performed by two anesthesiologists (Observer A and B) and one pediatric surgeon (Observer C). The scale consists of one positive item (M1: Perfect induction—no negative behavior, fear, or anxiety) and 10 negative behavior items (M2–M11). Each negative behavior was scored as “present = 1” or “absent = 0,” and the total score was calculated from M2–M11 items (range: 0–10). M1 is a mutually exclusive positive descriptor: when a perfect induction is observed, M1 is marked and all negative items are scored 0. M1 itself does not contribute to the total score and functions only as a clinical label for the ideal induction. All subsequent psychometric analyses (single‐factor confirmatory factor analysis, KR‐20, McDonald's omega, tetrachoric correlations, floor and ceiling effects, and item‐level inter‐observer reliability) were performed exclusively on the 10 negative items (M2–M11). M1 was used solely for descriptive purposes and was not included in the factor structure or reliability calculations of the total compliance score. Observations were conducted according to the standard anesthesia induction protocol, and the observers rated the behaviors independently.

#### 2.2.2 Inter‐Observer Reliability

Inter‐observer reliability for the total Induction Compliance Checklist score was assessed using the Intraclass Correlation Coefficient (InCC) with a two‐way random effects model. Both InCC (1, 2) (single rater) and InCC (2, 3) (average of three raters) were reported with 95% confidence intervals according to Koo and Li interpretation bands [17]. Item‐level reliability was evaluated using Fleiss' Kappa and Gwet's AC1 coefficients for the three observers. Construct validity was assessed through single‐factor confirmatory factor analysis (CFA) using the WLSMV estimator. Separate models were run for each observer, and the goodness‐of‐fit indices (CFI, TLI, RMSEA, and SRMR) were reported. Descriptive statistics are summarized as (mean and frequency). Data were analyzed using Excel, Python 3.12 and R 4.6.0.

#### 2.2.3 Internal Consistency

Internal consistency of the scale was examined using the Kuder–Richardson 20 (KR‐20) coefficient for each observer. In addition, McDonald's omega (ω) was calculated based on the tetrachoric correlation matrix. McDonald's omega is a factor‐analytic measure of internal consistency obtained under the assumption of a single‐factor model using tetrachoric correlations. KR‐20 and McDonald's omega values of ≥ 0.70 were considered acceptable and ≥ 0.80 good internal consistency.

#### 2.2.4 Item Analysis

For item analysis, the tetrachoric correlation matrix was calculated for each observer to evaluate inter‐item correlations. Additionally, floor effect (percentage of 0 scores) and ceiling effect (percentage of 1 scores) were determined for each item. Horn's parallel analysis was performed to determine the number of factors. This analysis aims to identify the number of factors to retain by comparing the real data with randomly generated datasets based on the tetrachoric correlation matrix.

## 3 Results

### 3.1 Content Validity

The I‐CVI values of the items ranged between 0.90 and 1.00 (Table 1). All items except “complete passivity, either rigid or limp” had an I‐CVI of 1.00; this item had an I‐CVI of 0.90. The Scale Content Validity Index (S‐CVI) was approximately 0.99, indicating that the content validity of the Turkish Induction Compliance Checklist was very high.

**TABLE 1:** Item content validity indices (I‐CVI) of the Induction Compliance Checklist (*n* = 10).

Item	I‐CVI	Decision
Perfect induction (no negative behaviors, fear or anxiety)	1.00	Accepted
M2. Crying, tears in eyes	1.00	Accepted
M3. Turns head away from mask	1.00	Accepted
M4. Verbal refusal, says “no”	1.00	Accepted
M5. Verbalization indicating fear or worry (“Where's mommy?” or “Will it hurt?”)	1.00	Accepted
M6. Pushes mask away with hands, pushes nurse/anesthetist with hands/ft	1.00	Accepted
M7. Covers mouth/nose with hands/arms or buries face	1.00	Accepted
M8. Hysterical crying, may scream	1.00	Accepted
M9. Kicks/flails legs/arms, arches back, and/or general struggling	1.00	Accepted
M10. Requires physical restraint	1.00	Accepted
M11. Complete passivity, either rigid or limp	0.90	Accepted

*Note:* The total score consists of items M2–M11.

### 3.2 Evaluation Procedure

#### 3.2.1 Descriptive Statistics

The demographic and clinical characteristics of the patients are presented in Table 2.

**TABLE 2:** Demographic and clinical characteristics of the patients (*n* = 184).

Characteristic	Value
Age (years)	4.24 ± 1.71 (1–7)
Median	4.0
Age Groups	
1–2 years	33 (17.9%)
3–4 years	67 (36.4%)
5–6 years	61 (33.2%)
7 years	23 (12.5%)
Gender	
Male	116 (63.0%)
Female	68 (37.0%)
Most Common Surgical Procedures	
Circumcision	56 (30.4%)
Inguinal hernia repair	31 (16.8%)
Adenotonsillectomy	18 (9.8%)
Orchiopexy	16 (8.7%)
Adenoidectomy	16 (8.7%)
Umbilical hernia repair	10 (5.4%)
Ventilation tube insertion	8 (4.3%)
Probing	7 (3.8%)
Other procedures	22 (12.0%)

### 3.3 Inter‐Observer Reliability

The InCC values calculated for the total Induction Compliance Checklist score among the three observers showed excellent agreement: InCC (1, 2) (single rater): 0.92 (95% CI: 0.90–0.94); InCC (2, 3) (average of 3 raters): 0.97 (95% CI: 0.96–0.98).

These values indicate excellent reliability according to Koo and Li (2016) criteria (17). Item‐level reliability analysis showed Fleiss' Kappa values ranging from 0.45 to 0.90 (generally 0.75–0.90 except for M11), while Gwet's AC1 values ranged from 0.81 to 0.97 (Table 3). These results confirm that the Turkish Induction Compatibility Checklist has excellent inter‐observer reliability.

**TABLE 3:** Item‐level inter‐observer reliability (Fleiss' Kappa and Gwet's AC1).

Item	Fleiss' Kappa	Gwet's AC1
M2 (Crying, tears in eyes)	0.90	0.92
M3 (Turns head away from mask)	0.80	0.81
M4 (Verbal refusal, says “no”)	0.83	0.83
M5 Verbalization indicating fear or worry, (“where is mommy?” or “will it hurt?”)	0.79	0.82
M6 (Pushes mask away with hands, pushes nurse Anesthetists with hands/ft)	0.82	0.83
M7 (Covers mouth/face with hands/arms or buries face)	0.75	0.85
M8 (Hysterical crying, may scream)	0.86	0.96
M9 (Kicks/flails legs/arms, arches back and/or general struggling)	0.88	0.95
M10 (Requires physical restraint)	0.89	0.96
M11 (Complete passivity, either rigid or limp)	0.45	0.97

*Note:* Overall, good‐to‐high agreement was observed across all components. Fleiss's Kappa density was low in M11 due to its low prevalence, but Gwet's AC1 remained high. 95% confidence intervals were calculated using the bootstrap method (2000 repetitions).

### 3.4 Construct Validity (Confirmatory Factor Analysis)

Confirmatory factor analysis (CFA) was performed to test the single‐factor structure of the scale using the WLSMV estimator for binary data (Table 4). Model fit varied across observers. The best fit was obtained for Observer C (CFI = 0.927, TLI = 0.907), approaching conventional thresholds for acceptable fit, whereas the indices for Observers A (CFI = 0.887, TLI = 0.854) and B (CFI = 0.781, TLI = 0.718) remained below recommended standards. The lower CFI and TLI values for Observers A and B are most likely attributable to the extreme floor effects and highly sparse binary data of the severe‐distress items (M7–M11), which destabilize the tetrachoric correlation matrix and inflate model misfit under WLSMV estimation. Overall, the single‐factor model received only partial support. Negative residual variances and negative loadings observed for some low‐prevalence items (particularly M9 and M10) are consistent with the sparsity of severe distress behaviors in this premedicated sample.

**TABLE 4:** One‐factor CFA fit indices.

Observer	*χ* ^2^ (sd)	CFI	TLI	RMSEA (90% GA)	SRMR
A	263.10 (35)	0.887	0.854	0.189 (0.168–0.210)	0.266
B	146.43 (35)	0.781	0.718	0.132 (0.110–0.154)	0.201
C	198.57 (35)	0.927	0.907	0.160 (0.139–0.182)	0.228

*Note:* The WLSMV estimator was used. Negative variance and negative factor loadings (M9 and M10) were observed in some items.

### 3.5 Internal Consistency

The KR‐20 and McDonald's omega (ω) values for the three observers are presented in Table 5. KR‐20 coefficients were low (0.49–0.52), while McDonald's omega values were moderate (0.66–0.82). These coefficients are lower than those typically expected for multi‐item psychological scales. The modest values are largely explained by the pronounced floor effects and highly skewed distributions of several items (particularly M8–M11), which reflect the infrequent occurrence of severe distress behaviors in this premedicated cohort. Under such conditions, classical internal‐consistency indices are known to be attenuated.

**TABLE 5:** Internal consistency coefficients of the induction compliance checklist.

Observer	KR‐20	McDonald's ω (tetrachoric)
A (Anesthesiologist)	0.52	0.82
B (Anesthesiologist)	0.49	0.66
C (Surgeon)	0.51	0.72

### 3.6 Item Analysis

Tetrachoric inter‐item correlation matrix analysis revealed generally low‐to‐moderate correlations between items (average inter‐item correlation: 0.09–0.12) (Table 6). The highest positive correlations were observed between M9 (kicking/struggling) and M10 (physical restraint) (0.89–0.94). This strong association supports the idea that both items reflect severe agitation and motor movement behaviors. The observation of negative correlations between some items (particularly M11 and other agitation items) is consistent with the scale's theoretical unidimensional structure. The mean correlation for item M11 was negative (−0.11), which is due to the item having a very low variance because of an excessive floor effect (96.7%–98.4%). The generally low‐to‐moderate inter‐item correlations indicate that the checklist items capture relatively distinct observable behaviors rather than highly redundant content. This pattern is expected in behavioral observation scales and does not contradict the presence of a single underlying latent factor of induction noncompliance, which was supported by the confirmatory factor analyses.

**TABLE 6:** Average tetrachoric inter‐item correlations of the Induction Compliance Checklist items (mean of 3 observers, *n* = 184).

Item	Average tetrachoric inter‐item correlation	Interpretation
M2 (Crying, tears in eyes)	0.03	Low
M3 (Turns head away from mask)	0.16	Moderate
M4 (Verbal refusal, says “no”)	0.20	Moderate
M5 (Verbalization indicating fear or worry, “where is mommy?” or “will it hurt?”)	0.19	Moderate
M6 (Pushes mask away with hands, pushes nurse Anesthetists with hands/ft)	0.18	Moderate
M7 (Covers mouth/face with hands/arms or buries face)	0.10	Low–Moderate
M8 (Hysterical crying, may scream)	0.20	Moderate
M9 (Kicks/flails legs/arms, arches back and/or general struggling)	0.08	Low
M10 (Requires physical restraint)	0.01	Very low
M11 (Complete passivity, either rigid or limp)	**−0.11**	Negative

*Note:* The bold value indicates an inverse relationship with the other checklist items. Mathematically, this distinct negative coefficient results from the item’s extreme floor effect (up to 98.4% zeros), which restricted variance.

When the floor and ceiling effects of the items were examined, the floor effect was found to be particularly pronounced in items M7 (74%–76%), M8 (85%–88%), M9 (81%–83%), M10 (84%–86%), and M11 (97%–98%) (Table 7). These results indicate that these negative behaviors are rare during pediatric anesthesia induction. The ceiling effect, on the other hand, remained at a low level overall and did not negatively affect the discriminatory power of the scale. These findings support the idea that, despite exhibiting a floor effect in the clinical population, the scale is acceptable in terms of capturing behavioral variation.

**TABLE 7:** Floor effect, ceiling effect, and average tetrachoric inter‐item correlations of the items (*n* = 184).

Item	Floor effect (%)	Ceiling effect (%)	Average tetrachoric inter‐item correlation
M2 (Crying, tears in eyes)	66.3–68.5	31.5–33.7	0.03
M3 (Turns head away from mask)	50.0–54.3	45.7–50.0	0.16
M4 (Verbal refusal, says “no”)	50.5–54.3	45.7–49.5	0.20
M5 (Verbalization indicating fear or worry, “where is mommy?” or “will it hurt?”)	60.9–65.2	34.8–39.1	0.19
M6 (Pushes mask away with hands, pushes nurse Anesthetists with hands/ft)	54.9–56.0	44.0–45.1	0.18
M7 (Covers mouth/face with hands/arms or buries face)	74.5–76.1	23.9–25.5	0.10
M8 (Hysterical crying, may scream)	85.9–88.0	12.0–14.1	0.20
M9 (Kicks/flails legs/arms, arches back and/or general struggling)	81.5–82.6	17.4–18.5	0.08
M10 (Requires physical restraint)	84.8–85.9	14.1–15.2	0.01
M11 (Complete passivity, either rigid or limp)	96.7–98.4	1.6–3.3	−0.11

*Note:* Average inter‐item correlation: 0.09–0.12 (range: −0.63–0.94).

### 3.7 Horn's Parallel Analysis

To examine the factor structure, Horn's parallel analysis was performed on the tetrachoric correlation matrix. The analysis initially suggested a possible 3‐factor solution. However, item M11 showed an extreme floor effect (96.7%–98.4%), rendering the tetrachoric correlation matrix nonpositive definite. As a result, the parallel analysis could not be completed reliably in several iterations, and the eigenvalue estimates were unstable. In light of this technical limitation, the theoretical unidimensional nature of the original ICC (a single continuum of induction compliance/noncompliance), and the acceptable fit of the single‐factor confirmatory factor analyses (particularly the best‐fitting model: CFI = 0.927, TLI = 0.907), a single‐factor model was retained (Figure 3). A multi‐factor solution was considered less appropriate because it would have produced poorly defined factors driven largely by the rarest items and would have reduced the clinical interpretability of the total score.

**FIGURE 3:**
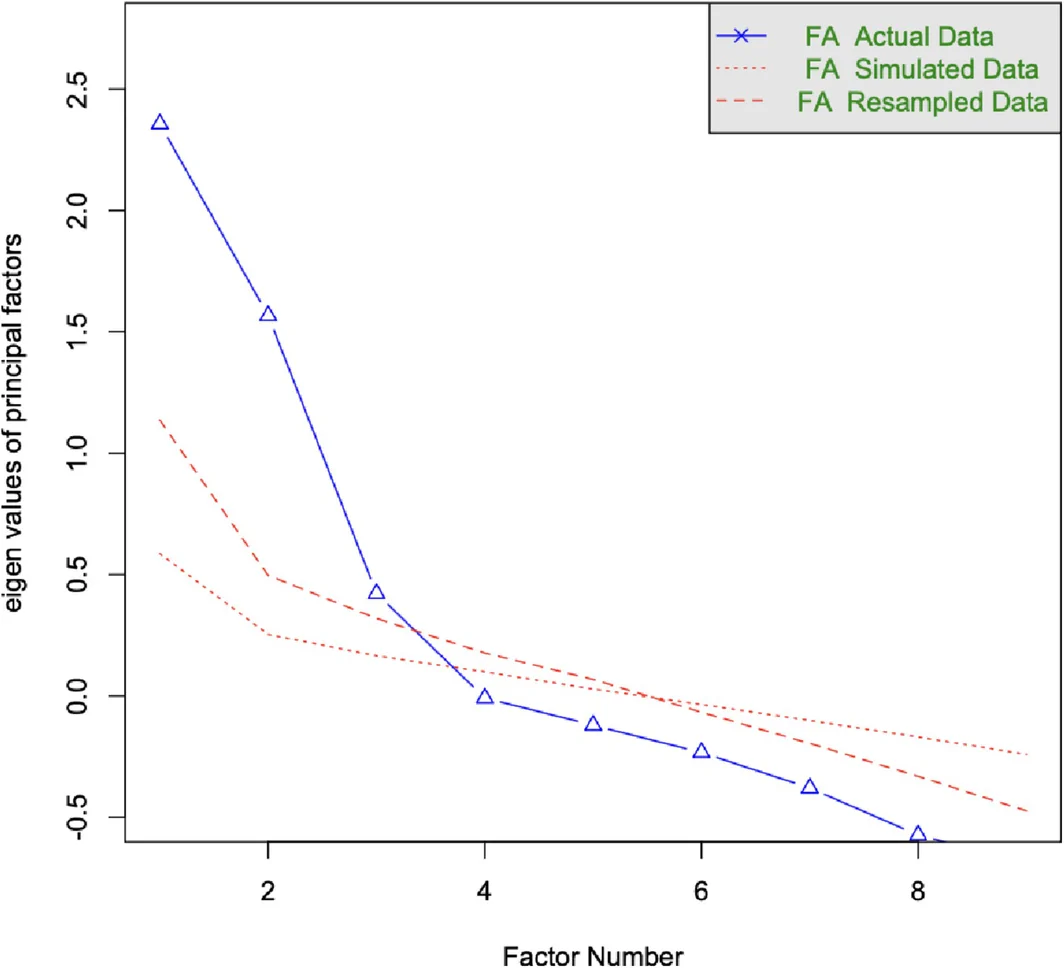
Factor‐model evaluation of the data.

## 4 Discussion

Under the conditions of this study, we showed that the newly developed T‐ICC is a reliable and valid tool to be used in the Turkish pediatric population. We further suggest that the rigorous process we undertook to develop the T‐ICC might possibly be used as a model in near‐future adaptation in clinical practice.

Induction of anesthesia is one of the most stressful moments for children undergoing surgery and frequently results in distress behaviors such as anxiety, crying, fear, and physical resistance [18]. These distress behaviors during induction are important predictors of negative postoperative outcomes, including emergence delirium, increased postoperative pain, negative behavioral changes, and decreased parental satisfaction [18]. It is therefore essential to measure these behaviors objectively, accounting for factors such as age, temperament, baseline anxiety, and past medical encounters [18]. While anxiety is primarily an internal emotional state often assessed through subjective tools (e.g., self‐report scales or the modified Yale Preoperative Anxiety Scale), compliance during induction represents observable behavioral responses to the specific procedural stimuli of mask induction. Measuring compliance provides direct, real‐time information about the child's actual cooperation with the anesthetic process, which may consist of diverse paths with preinduction anxiety levels.

Compliance with the ICC was assessed in this study immediately after premedication with oral midazolam administered 30 min before anesthesia induction. This approach provided procedure‐specific information on the child's actual behavioral response to the induction process itself. However, preoperative anxiety levels were not evaluated using standardized scales (e.g., mYPAS). Not evaluating preoperative anxiety levels with relevant scales might have resulted in lower ICC scores, as children with varying baseline anxiety could exhibit different compliance patterns.

Consequently, the moderate internal consistency values observed (KR‐20: 0.49–0.52; McDonald's ω: 0.66–0.82) are consistent with the characteristics of behavioral checklists in clinical populations where severe distress behaviors are relatively infrequent due to routine premedication. These levels are considered acceptable for observational tools in pediatric anesthesia research and do not undermine the scale's overall utility, especially given the excellent inter‐observer reliability and high content validity.

In the process of this study, the I‐CVI values evaluated with the opinions of ten experts ranged between 0.90–1.00, and the S‐CVI was at a very high level of approximately 0.99. In particular, the I‐CVI of 0.90 for the item “complete passivity, either rigid or limp” (M11) indicates that the experts judged this rarely observed behavior to be relevant and representative of the construct in the Turkish clinical context. In the study conducted in Spain, these values were reported to be partially low, and it was stated that a very small number of M11 items were detected as in our study [1]. High content validity indicates that the scale adapts well to the Turkish cultural context.

The InCC values for the total score obtained with three independent observers (0.92 and 0.97) show plausible agreement. A similar rate of inter‐observer reliability was also detected in the study conducted in Spain [1]. This result proves that the scale is objective and reproducible. The high Gwet's AC1 at the item level (0.81–0.97) supports practical reliability despite the low Kappa in M11 (due to low prevalence). Routine premedication with oral midazolam 0.5 mg/kg administered 30 min before induction, while reflecting standard clinical practice in many centers, likely attenuated the expression of severe distress behaviors. Consequently, items reflecting more intense negative responses (M7–M11) showed pronounced floor effects (74%–98%). The extreme rarity of M11 (“complete passivity, either rigid or limp”) produced the well‐recognized “kappa paradox”: Fleiss' Kappa was substantially lower than the high absolute agreement and Gwet's AC1 values because chance agreement on the dominant “absent” category is high when prevalence is near zero. This pattern is expected in observational behavioral checklists applied to routinely premedicated children and does not invalidate the scale. The excellent inter‐observer reliability of the total ICC score (InCC (1, 2) = 0.92; InCC (2, 3) = 0.97) and the consistently high Gwet's AC1 coefficients across items demonstrate that observers could still discriminate and agree on the behaviors that did occur. The moderate internal‐consistency coefficients are likewise consistent with a checklist in which several items are infrequently endorsed under usual clinical conditions.

Although the single‐factor confirmatory factor analyses showed acceptable approximate fit only for one of the three observers, the theoretical background of the original ICC, the excellent inter‐observer reliability of the total score, and the high content validity support the continued use of a single total compliance score in clinical practice. The suboptimal absolute fit indices for two observers are likely influenced by the extreme floor effects of several items and the resulting instability of the tetrachoric matrix—a known challenge when applying CFA to binary behavioral checklists with rare events.

Negative variances and loadings observed for some items (particularly M9 and M10) are attributable to the very low prevalence of these severe behaviors and are commonly reported in behavioral observation scales. Horn's parallel analysis initially suggested a possible 3‐factor solution; however, this result was limited by the extreme floor effect of item M11, which rendered the tetrachoric correlation matrix nonpositive definite and produced unstable estimates.

Taking into account this technical limitation, the theoretical conceptualization of the original ICC as a unidimensional continuum of induction compliance/noncompliance, and the acceptable fit of the single‐factor confirmatory factor analyses, a single‐factor model was retained. The low‐to‐moderate inter‐item correlations and the negative association of M11 (complete passivity, either rigid or limp) with active agitation items are considered compatible with a single underlying dimension rather than evidence of true multidimensionality. Overall, the findings support that the Turkish ICC primarily measures a single “compliance versus noncompliance” construct.

In the study by Ashbury et al., the 10‐item tool consistencies were found to be 0.34, and they stated that this showed an acceptable usage feature for all three observers [6]. The internal‐consistency coefficients obtained in this study (KR‐20: 0.49–0.52; McDonald's ω: 0.66–0.82) are modest. The low values are expected in behavioral checklists that contain multiple low‐frequency items: the heavy floor effects produce limited item variance and therefore attenuate classical reliability estimates such as KR‐20. McDonald's omega, which is derived from the factor model, was somewhat higher, supporting this explanation. Low‐moderate inter‐item correlations (average 0.09–0.12) show that the items measure different behaviors independently. The high correlation between M9‐M10 (0.89–0.94) supports that severe agitation behaviors are related to each other, while the negative correlation of M11 confirms that passivity is at the opposite pole from agitation. The high floor effect (especially 74%–98% in M7‐M11) reflects that severe negative behaviors are rare in the clinical population.

Nevertheless, the modest internal consistency does not negate the clinical usefulness of the Turkish ICC. The scale demonstrated excellent inter‐observer reliability for the total score, very high content validity, and the ability to quantify overall compliance in a straightforward manner. These properties remain valuable for both clinical monitoring and research applications in premedicated pediatric populations, even when traditional internal‐consistency metrics are reduced by item rarity.

Our findings support the ICC as a standard tool in pediatric anesthesia practice. The high reliability of the Turkish version will allow comparative studies to be conducted in different centers. This scale can be used to objectively evaluate the effectiveness of interventions such as premedication, parental presence, or distraction techniques. In addition, due to the high floor effect, the scale should be more sensitive in severe anxiety subgroups. With an easily created tool, making children's conditions predictable can make the environment a safer place for patients [6].

## 5 Limitations

The study has a number of methodological limitations. First, the sample was limited to ASA I‐III patients undergoing elective surgery, and emergency cases or children with neurological/developmental disorders were excluded. This may limit the generalizability of the scale's performance in more complex populations, and an expanded study including these patients can be conducted. Second, the floor effect is evident due to the low prevalence of some items (M8–M11), and this affected the internal consistency values. Negative variances in CFA also arise from this rarity.

Another important limitation is that oral midazolam premedication (0.5 mg/kg) was administered to all children 30 min before induction. Consequently, the psychometric properties of the Turkish ICC were evaluated exclusively in a premedicated pediatric population rather than in a general, non‐premedicated sample. The low prevalence of severe distress behaviors (and the resulting floor effects) may partly reflect the anxiolytic effect of premedication. The performance of the scale in children who do not receive premedication therefore remains to be established.

Finally, the single‐center design may not fully reflect cultural variations.

## 6 Conclusion

The Turkish Induction Compliance Checklist is a valid measurement tool with high content validity, excellent inter‐observer reliability, and acceptable internal consistency. It will contribute to the standardized and objective evaluation of children's behaviors during pediatric anesthesia induction. It is recommended for use in monitoring induction quality in clinical practice, evaluating intervention strategies, and in research protocols. With future multicenter studies, the scale's relationship with broader populations and long‐term outcomes can be examined. Finally, we suggest the psychometric process we undertook should be adopted in future translations of the ICC to other languages.

## Author Contributions

Y.A.S.: conceptualization, data curation, formal analysis, methodology, software, supervision, validation, writing‐original, writing‐review and editing. N.K.: conceptualization, data curation, methodology, validation. S.U.T: data curation, validation. C.Y.: data curation, validation. M.K.: data curation, validation. A.S.: formal analysis, methodology, software, supervision, validation, writing‐original, writing‐review and editing. ZNK: conceptualization, data curation, formal analysis, methodology, software, supervision, validation, writing‐original, writing‐review and editing.

## Funding

The authors have nothing to report.

## Conflicts of Interest

The authors declare no conflicts of interest.

## Data Availability

The data that support the findings of this study are available from the corresponding author upon reasonable request.
